# Repeatability, reproducibility and agreement of intraocular pressure measurement in rabbits by the TonoVet and Tono-Pen

**DOI:** 10.1038/srep35187

**Published:** 2016-10-12

**Authors:** Di Ma, Chong-Bo Chen, Jiajian Liang, Zhihao Lu, Haoyu Chen, Mingzhi Zhang

**Affiliations:** 1 Joint Shantou International Eye Center, Shantou University & the Chinese University of Hong Kong, Shantou 515041, China; 2 Shantou University Medical College, Shantou 515041, China

## Abstract

Tono-Pen and TonoVet have been used in rabbits to measure intraocular pressure (IOP) and investigate the effect of IOP lowering therapies. Therefore, their reliability and accuracy are very important and deserve careful evaluation. Our results showed that the with-subject deviation (*S*_w_) and intraclass correlation coefficient (ICC) of the TonoVet and Tono-Pen were 0.61 mmHg/0.83 mmHg and 0.97/0.94, respectively for intrasession repeatability. For intersession reproducibility, the Sw and ICC of TonoVet and Tono-Pen were 1.42 mmHg/1.66 mmHg and 0.73/0.67, respectively. For interoperator reproducibility, the *S*_w_ and ICC of the TonoVet and Tono-Pen were 0.72 mmHg/1.11 mmHg and 0.91/0.82 respectively. Both TonoVet and Tono-Pen underestimated the IOP measured by manometry. The regression function was: y = 0.8249x + 0.1011 and y =0.6881x + 2.2290 for TonoVet and Tono-Pen, respectively. Our study suggests that both TonoVet and Tono-Pen had excellent intrasession repeatability and inter-operator reproducibility, but good intersession reproducibility. Both TonoVet and Tono-Pen correlated well with manometry, but underestimated the manometric IOP with presence of fixed and proportional biases. These factors should be considered when measuring IOP with Tono-Pen or TonoVet in rabbit eyes.

Intraocular pressure (IOP) is the fluid pressure of aqueous humor inside the eyeball. It is an essential parameter for diagnosis, follow up and treatment of glaucoma and other ocular diseases. Currently, several different methods have been developed to measure IOP, including indentation^1^, applanation^2^, rebound^3^, ocular response analyzer^4^ and dynamic contour tonometery^5^. Generally, these tonometers are designed for human use, or more precisely for use in white populations^6^,^7^. However, novel therapies aiming at lowering intraocular pressure must undergo their initial tests in animal models and demonstrate their effectiveness prior to clinical trials. Many investigations were performed on non-primate animal models, among which, rabbits are becoming a popular model, for evaluation of new drugs and surgical procedures for glaucoma, owing to their resemblance to the human eye in size and a better understanding of their eye anatomy and physiology. However, there are distinct differences between human and rabbit eyes in terms of corneal thickness and composition^8^. Therefore, it is of great essence and importance to evaluate the precision and accuracy of IOP measuring devices to validate whether they are suitable and reliable for estimation of IOP in rabbits.

Currently, the portable and non-invasive tonometers, including TonoVet and Tono-Pen, are widely used. The TonoVet, based on rebound tonometry, is equipped with a magnetic probe that, when electromagnetically propelled against the cornea, produces a resultant rebound from the cornea to induce a voltage change, which is converted an electric signal representing the IOP^9^. In contrast, the Tono-Pen is based on the principle of applanation tonometry, and has been used for a wide range of species. It measures IOP indirectly by applying the force needed to flatten a certain area of cornea surface, which can be converted into a pressure reading that is equivalent the pressure in the eye^10^.

Previous articles^8^,^11^,^12^,^13^,^14^,^15^,^16^ have reported the use of TonoVet or Tono-Pen in rabbits. The accuracy of Tonometer has been measured in terms of its agreement with direct measurement of intracameral pressure^12^. However, their results are inconsistent. Furthermore, repeatability and reproducibility are important parameters to evaluate the variability of a measurement. To the best of our knowledge, there is no such a report comparing the repeatability and reproducibility of TonoVet and Tono-Pen.

This study was undertaken to assess the intrasession repeatability, intersession and inter-operator reproducibility of the Tono-Pen and TonoVet in measurement of IOP in rabbit eyes. Additionally, the accuracy of the tonometers was determined by comparing the measured IOPs, taken via Tono-Pen and TonoVet, with IOPs obtained manometrically.

## Results

### Intrasession Repeatability

Table 1 presents the mean IOP values, within-subject standard deviation (*S*_*w*_), coefficient of variation (CVw), precision and intraclass correlation coefficient (ICC) values for intrasession repeatability when using the TonoVet and Tono-Pen. The *S*_w_, CVw, precision and ICC values for the TonoVet were 0.61 mmHg, 5.50%, 1.19 mmHg and 0.97, respectively. Likewise, the *S*_w_, CVw, precision and ICC values for the Tono-Pen were 0.86 mmHg, 7.72%, 1.63 mmHg and 0.94, respectively. The very high ICC values (ICC = 0.97 for TonoVet while ICC = 0.94 for Tono-Pen) together with quite low *S*_w_ and small precision range indicated excellent intrasession repeatability of TonoVet and Tono-Pen for IOP measurement. Furthermore, the IOP on average measured by TonoVet (11.02 ± 1.65 mmHg) was marginally different from that by Tono-Pen (10.78 ± 1.69 mmHg) (*p* = 0.026, Student’s paired *t*-test).

### Intersession and interoperator reproducibility

Tables 2 and 3 show the mean IOP values, *S*_w_, CVw, precision and ICC values for intersession and interoperator reproducibility, respectively. Both TonoVet and Tono-Pen have higher values of *S*_w_, CVw and precision in intersession reproducibility than intrasession variation. In contrast, regarding interoperator reproducibility, TonoVet and Tono-Pen possess low *S*_w_ but small to moderate CVw and precision. The moderate intersessional ICC values (ICC = 0.73 and 0.67 for TonoVet and Tono-Pen, respectively) and high interoperator ICC values (ICC = 0.91 and 0.82 for TonoVet and Tono-Pen, respectively) indicated that there was good intersession reproducibility and excellent interoperator reproducibility of TonoVet and Tono-Pen for IOP measurements. It should be noted that the average IOP obtained by TonoVet (11.06 ± 1.16 mmHg) in intersession reproducibility marginally differed from that by Tono-Pen (10.80 ± 1.19 mmHg) (*p* = 0.047, Student’s paired *t*-test) while in interoperator reproducibility the mean IOP read by TonoVet (11.06 ± 1.62 mmHg) was not significantly different by Tono-Pen (10.80 ± 1.69 mmHg) (*p *= 0.099, Student’s paired *t*-test).

### Diurnal change of IOP

**Figure 1:**
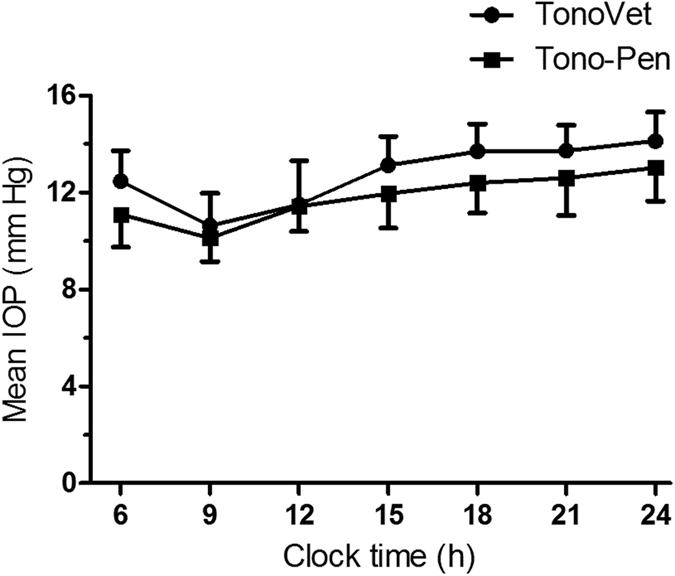
Intraocular pressure (IOP) measured by TonoVet and Tono-Pen over a 24-hour period. Symbols represent mean ± SD.

The profile of the mean rabbit IOP over a 24-h period measured by TonoVet and Tono-Pen is shown in Fig. 1. The fluctuation profile of the IOP obtained with TonoVet and Tono-Pen showed a similar pattern; the IOP gradually decreased from 6 AM to 9 AM, and then appeared to gradually increase from 9 AM to 12 PM. The lowest IOP was recorded at 9 AM (10.63 and 10.13 mmHg for TonoVet and Tono-Pen, respectively) while the highest one was recorded at around 12 PM (14.13 and 13.03 mmHg for TonoVet and Tono-Pen, respectively). Generally, the IOP in the daytime was lower than that in the nighttime.

### Agreement with manometry

**Figure 2:**
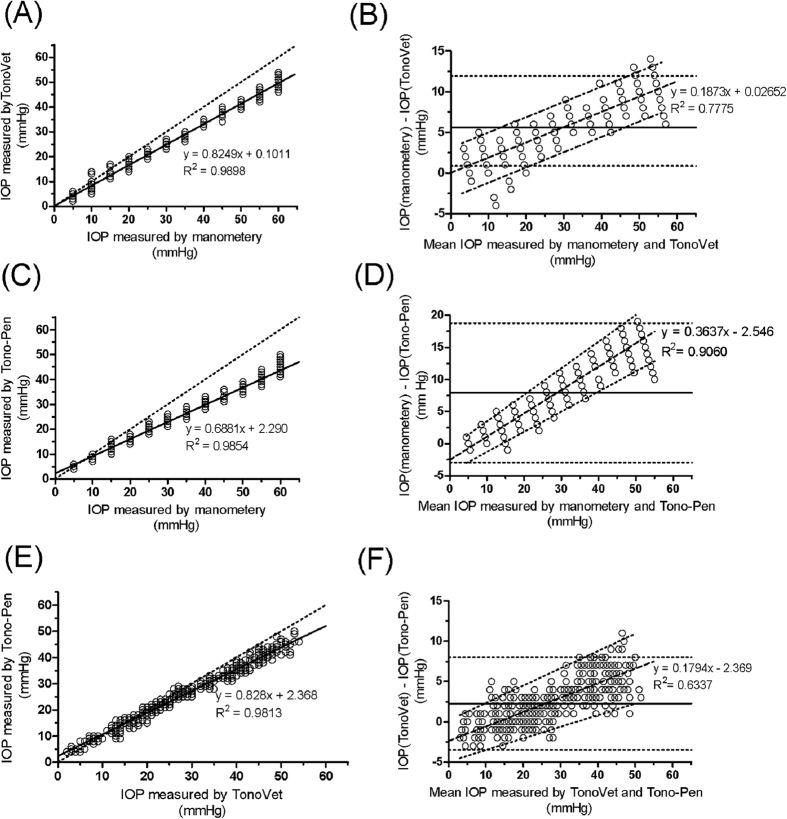
Correlation and Bland-Altman plots of agreement between manometry vs. TonoVet and Tono-Pen. (**A**) Correlation between manometry and TonoVet. Solid and dotted lines represent best-fit regression line and the ideal regression line (i.e. y = x), respectively. (**B**) Correlation between manometry and TonoVet. The solid and dash-dot lines represent the regression line of difference on average and regression-based 95% limit of agreement, respectively. (**C**) Correlation between manometery Tono-Pen. (**D**) Correlation between manometry and Tono-Pen. (**E**) Correlation between Tono-Pen and TonoVet. (**F**) Agreement between Tono-Pen and TonoVet.

There was high correlation between real IOP and IOP measured with either the TonoVet (r^2^ = 0.9949, *p* < 0.0001) or Tono-Pen (r^2^ = 0.9927, *p* < 0.0001). However, significant difference was observed between the TonoVet IOP value (*p *= 0.000, Student’s paired *t*-test) and the Tono-Pen IOP value (*p *= 0.000, Student’s paired *t*-test) vs. the manometric IOP value at all levels except at 5 mmHg for Tono-Pen. Both the TonoVet and the Tono-Pen underestimated the real IOP and the difference increased with the increase in IOP (Fig. 2A–D). The 95% regression-based limits of agreement are shown in Table 4.

Except at pressure between 5 and 15 mmHg, IOP read by TonoVet was significantly higher than that by Tono-Pen (*p* ≤ 0.005, independent *t*-test) (Table 5). The correlation between the TonoVet and Tono-Pen reading (r^2^ = 0.9906, *p* < 0.0001) was high (Fig. 2E). The mean difference in IOP measured by TonoVet and Tono-Pen was 2.29 mmHg, being distinguishable from zero (*p *= 0.000, one-sample *t*-test), which indicated that there was a fixed bias. Additionally, a Bland-Altman test revealed a proportional bias, since the slope of regression of differences on averages was distinguishable from zero (*p *< 0.0001). Nevertheless, the TonoVet apparently tended to give a higher IOP relative to Tono-Pen at manometric pressure more than 20 mmHg and tended to give a lower IOP relative to Tono-Pen at manometric pressure no more than 5 mmHg.

## Discussion

In this study, we assessed the repeatability and reproducibility of TonoVet and Tono-Pen for IOP measurement in rabbits, and determined their agreement with manometric IOP. We found that IOP measurements with TonoVet and Tono-Pen have excellent intrasession repeatability, as evidenced by a low variability (*S*_*w*_ ≤ 0.83 mmHg) and small precision range within 1.63 mmHg, as well as high ICC values ranging between 0.94 and 0.97. Furthermore, the two devices had excellent interoperator reproducibility as indicated by low variability (*S*_*w *_≤ 1.11 mmHg) and high ICC values between 0.82 and 0.91. Both devices displayed good intersession reproducibility, as demonstrated by medium variability (*S*_*w *_≤ 1.66 mmHg) with wider precision range beyond 2.78 mmHg and moderate ICC values varying from 0.67 to 0.73. The lower intersession reproducibility may be due to the variation of IOP measured on different days.

Our study is the first one to compare the repeatability, intersession and interoperator reproducibility of TonoVet and Tono-Pen in rabbit. Our results found that TonoVet was superior to Tono-Pen in terms of intrasession repeatability, intersession and interoperator reproducibility as indicated by less variability and higher ICC, suggesting that TonoVet was more precise than Tono-Pen. Nevertheless, our repeatability result of Tono-Pen was better than that reported by Acosta *et al*. who found that the ICC value for interobserver varied from 0.48 to 0.69 at normal rabbit IOP levels^8^. The difference might be likely attributed to their small sample size with only 4 rabbit eyes being measured.

The comparison of TonoVet and Tono-Pen against the manometric method for measuring IOP in rabbits has been investigated previously^8^,^11^,^12^,^13^,^14^,^15^,^16^. Lim *et al*. reported that Tono-Pen underestimates the actual IOP and is apt to error at high IOP levels, but it has been found to be the most accurate tonometer among the Tono-Pen, Perkins and pneumatonometer^11^. Acosta *et al*. found that Tono-Pen underestimated rabbit IOP at all pressure ranges from 5 to 50, and the low prediction was more notable at high pressures^8^. Zhang *et al*. reported that TonoVet tended to consistently display lower IOP values than manometer values in all pressure ranges, regardless of using a ‘d’ (dogs or cats) or ‘p’ (other species) mode of calibration^15^. In agreement with previous studies, our findings indicated that both TonoVet and Tono-Pen consistently underestimated the actual IOP except at 5 mmHg for the Tono-Pen and the underestimation increased with the increase in IOP. The increasing inaccuracy might be attributed to the measurement error, which is increased for both devices as the pressure increases. Kalesnykas *et al*. reported that Tono-Pen overestimated IOP at reservoir pressure no more than 10 mmHg and underestimated IOP otherwise^12^. The inconsistency might be explained by the fact that their data were based on small sample size (n = 2).

To assess whether TonoVet and Tono-Pen had a similar degree of low prediction, we determined the difference of TonoVet and Tono-Pen measurements. As shown in Table 4, TonoVet had a tendency to measure a significantly higher IOP (*p =* 0.000, independent *t*-test) relative to Tono-Pen at real pressure greater than 20 mmHg, and gave a markedly lower IOP (*p* *=* 0.000, independent *t*-test) relative to Tono-Pen at pressure lower than 5 mmHg. However, at a pressure level between 10 and 15 mmHg, TonoVet was comparable with Tono-Pen in IOP estimation. These results suggest that Tono-Pen could be more accurate at IOP no more than 5 mmHg, whereas TonoVet could be more accurate at IOP higher than 20 mmHg. Overall, TonoVet was significantly more accurate than Tono-Pen, which suggested that TonoVet could be a more predictable device for evaluation of effectiveness of drugs or surgical treatments in rabbit eyes.

Differences in measurements can be attributed not only to the instruments and topical anesthesia, but also to the fluctuation of IOP in rabbit’s eyes. In our study, the mean IOP gradually decreased from 6 AM to 9 AM, and then gradually increased from 9 AM to 12 PM. Our results are in good agreement with a previous study, conducted in rabbits, which showed that the IOP during the daylight period (7 AM to 7 PM) was lower than that during the night (8 PM to 6 AM)^17^. The fluctuation pattern of IOP is likely linked to the variation of circadian rhythms and β-adrenergic systems during day-night alternation, which has an impact on the formation and regulation of aqueous humor^13^,^18^,^19^.

We recognized some limitations of the current study. First, measurement of IOP using rebound or applanation may be affected by the biomechanical characteristics of cornea. The tonometers were designed based on the characteristics of normal cornea. Therefore, we need to be careful when proposing the use of these instruments for assessing the treatment efficacy parameters in animal models of glaucoma where the corneal status of these animals remains unknown. Secondly, the performance of IOP measurement depends on the operator. Proper operation and experience are needed in clinical practice of IOP measurement.

In summary, we find that both TonoVet and Tono-Pen exhibit good precision in terms of excellent intrasession repeatability, excellent interoperator reproducibility and good intersession reproducibility. Additionally, progressive underestimation of the true IOP by TonoVet and Tono-Pen was observed with fixed and proportional biases, but overall the TonoVet was superior to Tono-Pen in both precision and accuracy. Altogether, the hand-held Tono-Pen could be expected to be a reliable tonometer for measurement of normal rabbit IOP, while the TonoVet could be applied for the measurement of IOP in both normal and hypertensive eyes.

## Materials and Methods

### Animals

The Ethics Committee for Animal Study of the Joint Shantou International Eye Center approved this study. The experiments were designed and conducted in accordance with the Association of Research in Vision and Ophthalmology Statement for the Use of Animals in Ophthalmic and Visual Research. Thirty female New Zealand albino rabbits were used with an average age of 5 months (range, 4–6 months) and mean weight of 2.8 kg (range, 2.5 to 3.1 kg). Rabbits were handled gently and were required to be calm with gentle touch at least 3 minutes before IOP measurement. If any sign of stress was found, IOP measurements were postponed for at least another 3 minutes. We used the right eyes only for IOP measurement to avoid bias due to bilateral correlation. All the measurement was performed between 8 to 10 am except in the diurnal study.

### IOP measurements by TonoVet

IOP measurements were first performed with the TonoVet (Helsinki, Finland). Since there was no calibration mode programmed for rabbit in the TonoVet, a dog calibration mode was chosen instead. After securing the rabbit, the TonoVet was brought near the rabbit’s eye and fixed at the rabbit’s nose. The central groove of the tonometer was kept in a horizontal position and the distance was maintained at 4–8 mm between the tip of the probe and the cornea. The measurement button was pressured lightly to enable the tip of the probe to hit the central cornea. Six consecutive measurements were taken and the resulting IOP was displayed.

### IOP measurements by Tono-Pen XL

The Tono-Pen XL (Reichert, Depew, NY) was equipped with an Ocu-Film tip cover and calibrated as instructed in the manufacturer’s manual prior to the first use each day. A drop of Alcaine ophthalmic solution (Alcon, Fort Worth, TX) was instilled onto the rabbit’s eye before the measurement. To reduce hand-held tonometer movement, the elbow of the operator was braced on the table for stability. The Tono-Pen transducer was held perpendicular to and within 1/2 an inch of the rabbit’s cornea. After pressing the operator’s button to initiate an IOP measurement and following the beep tone, the probe tip was touched lightly with the central cornea without indentation and then withdrawn. After four valid measurements, a final beep sounded and the averaged measurement displayed on the LCD, along with the single bar denoting statistical reliability. Measurements were repeated until the displayed coefficient of variation was less than 5%. The Ocu-Film tip cover was replaced before using the Tono-Pen XL unit on another rabbit.

### Repeatability and reproducibility study

The precision of tonometry was characterized by its repeatability and reproducibility. Repeatability is defined as the intrasession variability for repeated measurements, which are performed by the same operator using the same instrument during a short period. Reproducibility refers to the variability in repeated measurements in which time or the operator is varied, characterized as intersession variability and interoperator variability, respectively. According to the assumption^20^ that the 95% confidence interval of within-subject standard deviation (*S*_*w*_) to be estimated within 15% of *S*_w_, 1.96
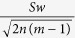
= 15% × *S*_w_, sample size can be calculated using the following formula: *n* = 1.96^2^/[2(*m*-1) × 0.15^2^], where *n* and *m* represent as the number of subjects and observations (*m* = 4, *n* = 30 on this study), respectively. When measuring, IOP of the same eyes was determined with TonoVet and subsequently with Ton-Pen by two respective blinded operators consecutively for 4 days. The operators were blind to the reading of the other operator. In the repeatability analysis, four measurements from each operator measured every day were used while in the reproducibility analysis, the second measurement from each operator on each day was used. Parameters were calculated with Excel and SPSS, and included the intrasession, intersession and interoperator within-subject standard deviation (*S*_w_), precision (repeatability coefficient) (1.96 × *S*_w_), coefficient of variation (CVw) (100 × *S*_w_/overall mean) and intraclass correlation coefficient (ICC). ICC values greater than 0.75 were considered as excellent reproducibility, between 0.40 and 0.59 as fair, between 0.6 and 0.75 as good reproducibility, and lower than 0.4 poor reproducibility^8^.

### Diurnal change of IOP

Since intraocular pressure is dynamic and fluctuates with the change of physiological state, IOP in 10 rabbits of both eyes was measured every 3 hours, beginning at 6 AM and ending at midnight, by a single operator under same conditions as stated in repeatability measurement.

### Agreement with manometric measurements

Sample size was calculated for the agreement study according to the formula *n* ≥ 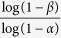
21, where *n*, *α* and *β* represent the sample size, discordance rate and tolerance probability, respectively (When *α* *= *0.15 and *β* = 80%*, n* ≥ 10). The experiment was performed under general anesthesia using ketamine (30 mg/kg) and xylazine (5 mg/kg) intramuscularly. Two 25-gauge needles were inserted into the anterior chamber of the right eye via a self-sealing limbal incision at the 3 and 9 o’clock positions. One of them was connected via polyethylene tubing to a bottle filled with balanced salt solution under open stopcock condition, while the other was connected to a vertical water column for measurement of IOP. The height of the balanced salt solution bottle was vertically altered to achieve various IOPs, ranging from 60 to 5 mmHg by 5-mmHg decrements, based on the reading of the water column. When the reading of the water column reached a stable value for more than 2 minutes, four sets of IOP readings were taken blindly with TonoVet, and subsequently with Tono-Pen, at each bottle height. IOP readings of the two tonometers at each pressure level were compared with the actual IOP to determine the accuracy. Pearson’s correlation and linear regression analyses were used to determine a best-fit line for assessing the variation of measurement error. Bland-Altman bias plots^22^,^23^ were drawn to evaluate the level of agreement between IOP values measured by the two tonometers and manometer.

## Additional Information

**How to cite this article**: Ma, D. *et al*. Repeatability, reproducibility and agreement of intraocular pressure measurement in rabbits by the TonoVet and Tono-Pen. *Sci. Rep.* **6**, 35187; doi: 10.1038/srep35187 (2016).

## Figures and Tables

**Table 1:** Intrasession repeatability of TonoVet and Tono-Pen.

Day	Tonometer	Operators	Mean ± SD (mmHg)	Sw (mmHg)	CVw (%)	Precision	ICC
1	TonoVet	Operator 1	11.65 ± 1.76	0.69	5.93	1.35	0.96
		Operator 2	11.61 ± 1.27	0.70	6.01	1.37	0.93
	Tono-Pen	Operator 1	11.05 ± 1.85	0.91	8.26	1.79	0.94
		Operator 2	11.61 ± 1.38	1.07	9.24	2.25	0.87
2	TonoVet	Operator 1	11.13 ± 1.81	0.55	4.95	1.08	0.98
		Operator 2	10.90 ± 1.66	0.53	4.88	1.04	0.97
	Tono-Pen	Operator 1	11.57 ± 1.65	0.82	7.12	1.61	0.94
		Operator 2	10.90 ± 1.85	0.78	7.14	1.19	0.96
3	TonoVet	Operator 1	10.82 ± 1.62	0.51	4.72	1.00	0.98
		Operator 2	10.80 ± 1.79	0.57	5.30	1.12	0.98
	Tono-Pen	Operator 1	10.28 ± 1.59	0.73	7.14	1.44	0.95
		Operator 2	10.80 ± 1.49	0.77	7.16	1.17	0.94
4	TonoVet	Operator 1	10.95 ± 1.42	0.50	4.57	0.98	0.97
		Operator 2	10.93 ± 1.55	0.58	5.35	1.15	0.97
	Tono-Pen	Operator 1	10.34 ± 1.47	0.79	7.59	1.54	0.93
		Operator 2	10.93 ± 1.61	0.82	7.54	1.33	0.94
Average	TonoVet		11.02 ± 1.65	0.61	5.50	1.19	0.97
	Tono-Pen		10.78 ± 1.69	0.83	7.72	1.63	0.94

Sw: with-subject deviation; CVw: coefficient of variation; ICC: intraclass correlation coefficient.

**Table 2:** Intersession reproducibility of TonoVet and Tono-Pen.

Tonometer	Mean ± SD (mmHg)	Sw (mmHg)	CVw (%)	Precision	ICC
TonoVet	11.06 ± 1.16	1.42	12.84	2.78	0.73
Tono-Pen	10.80 ± 1.24	1.66	15.38	3.25	0.67

Sw: with-subject deviation; CVw: coefficient of variation; ICC: intraclass correlation coefficient.

**Table 3:** Interoperator reproducibility of TonoVet and Tono-Pen.

Tonometer	Mean ± SD (mmHg)	Sw (mmHg)	CVw (%)	Precision	ICC
TonoVet	11.06 ± 1.62	0.72	6.50	1.41	0.91
Tono-Pen	10.80 ± 1.69	1.11	10.30	2.18	0.82

Sw: with-subject deviation; CVw: coefficient of variation; ICC: intraclass correlation coefficient.

**Table 4:** Bland-Altman 95% limits of agreement for manometry vs. TonoVet and Tono-Pen.

Agreement	Mean difference (mmHg)	*p* value*	Fixed bias	*p* value#	Proportional bias	95% limit of agreement
Manometry vs. TonoVet	5.63	0.000	Yes	<0.0001	Yes	−3.0566 + 0.1873x to 3.1096 + 0.1873x
Manometry vs. Tono-Pen	7.92	0.000	Yes	<0.0001	Yes	−4.4230 + 0.3136x to −0.6690 + 0.4138x
TonoVet vs. Tono-Pen	2.29	0.000	Yes	<0.0001	Yes	−4.9397 + 0.1441x to 0.2017 + 0.2147x

^*^One-sample t-test.

^#^Pearson correlation.

**Table 5:** Comparison of TonoVet and Tono-Pen versus actual IOP between 5 and 60 mmHg.

Actual IOP (mmHg)	Bottle height (cm)	TonoVet		Tono-Pen		TonoVet vs. Tono-Pen
		Mean + SD	*p*	Mean + SD	*p*	*p*
5	6.8	3.53 + 0.97	0.000*	4.89 + 0.71	0.353	0.000*
10	13.6	8.30 + 2.08	0.000*	8.90 + 0.84	0.000*	0.097
15	20.4	12.85 + 1.49	0.000*	12.88 + 1.40	0.000*	0.939
20	27.2	17.10 + 1.28	0.000*	16.20 + 1.18	0.000*	0.002*
25	34.0	20.98 + 1.10	0.000*	20.23 + 1.21	0.000*	0.005*
30	40.8	24.58 + 1.06	0.000*	23.45 + 1.28	0.000*	0.000*
35	47.6	28.60 + 1.30	0.000*	26.73 + 1.24	0.000*	0.000*
40	54.4	33.43 + 0.96	0.000*	29.88 + 1.18	0.000*	0.000*
45	61.2	37.75 + 1.26	0.000*	32.80 + 1.30	0.000*	0.000*
50	68.0	41.05 + 1.01	0.000*	36.23 + 1.37	0.000*	0.000*
55	74.8	44.93 + 1.56	0.000*	39.73 + 1.83	0.000*	0.000*
60	81.6	49.78 + 2.03	0.000*	43.88 + 2.28	0.000*	0.000*

SD: standard deviation.
